# Self-Weighing Behaviors of Diverse Community-Dwelling Adults Motivated for a Lifestyle Change

**DOI:** 10.3390/ijerph19095242

**Published:** 2022-04-26

**Authors:** Yoshimi Fukuoka, Yoo-Jung Oh

**Affiliations:** 1 Department of Physiological Nursing, University of California, San Francisco, San Francisco, CA 94143, USA; 2 Department of Communication, University of California, Davis, Davis, CA 95616, USA; yjeoh@ucdavis.edu

**Keywords:** body mass index, obesity, self-monitoring, self-report, anthropometric measurements, self-weighing

## Abstract

We aimed to understand adults’ self-weighing behaviors and explore significant predictors of body mass index (BMI) accuracy based on self-reported height and weight in a diverse sample of community-dwelling adults. **Methods:** In this cross-sectional study, 531 adults participating in a physical activity program or a weight loss program were analyzed. Participants’ self-reported and objectively measured weight, height, weight scale ownership, self-weighing behaviors, and medical history were collected. **Results:** The mean age (standard deviation) was 50.0 (12.0) years with a range of 24 to 78 years. Out of 531 participants, 455 (85.7%) were women. The study population was diverse (58.9% non-White). In total, 409 (77.0%) participants had a weight scale at home, but only 222 (41.8%) weighed themselves at least once a week. The weight and BMI underestimation became much more significant as the participant’s weight increased (*p* ≤ 0.001). Employment status, high cholesterol, and low objectively measured weight were significant predictors of self-reported BMI accuracy after controlling for potential confounding factors (*p* < 0.05). Interestingly, ownership of a home weight scale and the frequency of self-weighing behavior were not significantly associated with the accuracy of self-reported BMI (*p* > 0.05). **Conclusion:** The accuracy of the participants’ BMI, based on self-reported height and weight, was significantly associated with employment status, high cholesterol, and low objectively measured weight, suggesting that BMI accuracy depends on multi factors.

## 1. Introduction

The prevalence of obesity among adults is increasing at an alarming rate, rising from 30.5% in 2000 to 42.4% in 2018 in the United States [1]. In addition, obesity is a major risk factor for chronic illness. Self-reported weight and height are often used to estimate body mass index (BMI) in national surveys and epidemiologic studies to monitor the effect of the obesity epidemic over time [2]. Using self-reported weight and height is cost-effective and feasible, but is likely to have social desirability and recall biases. Several systematic review reports demonstrate that adults underestimate their body weight and overestimate their height, resulting in lower BMI [3,4,5,6]. However, only a few systematic reviews have described the characteristics associated with self-reported weight and height accuracy. 

Self-weighing can be an essential contributing factor to one’s accurate estimation of weight. Self-monitoring, observing, and recording weight over time are critical elements of weight management and weight loss [7,8,9,10]. Weight tracking over a period facilitates self-regulation, thereby allowing for frequent, small adjustments such as increased physical activity and reduced caloric intake. Several systematic reviews of self-weighing have reported that frequent self-weighing is associated with improved weight loss and maintenance [10,11]. Recent literature supports the notion that increased self-weighing does not lead to adverse psychological effects, and consistent daily self-weighing was positively perceived by the participants who found it easy to remember as a routine practice [11,12]. However, most studies focus on self-weighing behavior after weight-loss interventions. Little is known about the self-weighing behavior of individuals who are highly motivated to improve their lifestyle by increasing physical activity and/or losing weight before initiating these programs. 

Individuals who want to modify their lifestyle can easily engage with regular self-weighing if they have a scale at home. Baseline information on self-weighing behaviors and the prevalence of scale ownership can be useful when designing a lifestyle modification program. To advance scientific knowledge, we systematically collected information on self-weighing behaviors and self-reported and objectively measured weight, height, and BMI in community-dwelling adults interested in participating in a lifestyle modification program. This study aimed to describe self-weighing behaviors and identify significant predictors of BMI accuracy in a diverse sample of community-dwelling adults.

## 2. Materials and Methods

### 2.1. Design, Setting, and Sample

In this cross-sectional study, adults participated in a physical activity program or a weight loss (physical activity and diet) program at the University of California, San Francisco (UCSF) [13,14,15]. In total, 1613 potential participants interested in one of our studies contacted a trained research staff member and initiated a telephone screening. Of those, 1060 were excluded due to not meeting eligibility criteria, not completing a telephone screening, or losing interest in study participation. The remaining 553 potential participants completed an in-person screening and baseline visit. However, 22 out of 553 potential participants were excluded from this analysis due to missing at least one key variable. We analyzed 531 participants in the present study. Participants were recruited through online and media advertisements, community events, and the posting of study flyers in clinics, hospitals, local businesses, and community centers in the San Francisco Bay Area, California. The UCSF institutional review board approved the protocols, and written consent was obtained from all participants. The inclusion criteria were as follows: physically inactive at work and during leisure time based on the Stanford Brief Activity Survey, which consists of five categories (i.e., inactive, light activity, moderate activity, hard activity, and very hard activity) [16], and intention to become physically active; being overweight and wanting to lose weight; aged 20 years and older; and the ability to speak and read English. The exclusion criteria were: known medical or physical conditions that require special attention in an exercise or weight loss program; pregnant or having given birth during the preceding 6 months; severe hearing or speech disorders; history of eating an disorder; current substance abuse; currently participating in lifestyle modification programs or research studies that may confound the study results; or mild cognitive impairment as determined by the Mini-Cog test [17,18]. 

### 2.2. Procedures and Measures

After obtaining verbal consent, the trained research staff screened potential participants by phone for a preliminary screening of eligibility. During the telephone screening, the trained research staff assessed the participants’ self-reported weight, height, weight scale ownership, self-weighing behaviors, and medical history. We also asked the following question: “Do you know your BMI, which is calculated by your weight and height?” If the answer was “yes”, participants were asked to report their estimated BMI. If the answer was “no”, participants were asked to select the most appropriate BMI category among the four classifications: underweight (BMI < 18.5 kg/m^2^), normal weight (BMI ≥ 18.5 to <25 kg/m^2^), overweight (BMI ≥ 25 to <30 kg/m^2^), and obese (BMI ≥ 30 kg/m^2^). Potential participants who met the preliminary eligibility criteria were invited to attend a screening and baseline visit, and they were asked to review the consent form before their visit.

At the beginning of the screening and baseline visit, the trained research staff explained the study again and obtained written consent from all participants. Participants were asked to complete the sociodemographic form and other questionnaires. In the physical examination room, participants changed into hospital gowns and removed their shoes for anthropometric measurements. The research staff measured participants’ height and weight twice using a Seca portable stadiometer (Seca Deutschland, Hamburg, Germany) and a Tanita WB-110 digital electronic scale (Tanita Corporation of America, Inc., Arlington Heights, IL, USA), respectively. The average of the two readings was used to calculate BMI (i.e., objectively measured BMI). 

### 2.3. Analytic Strategy

Descriptive statistics were used to describe the participants’ sociodemographic and medical information, self-weighing, and knowledge of their BMI. Multiple one-way analyses of variance investigated whether individuals in quartiles of objectively measured weight groups would significantly differ in terms of objectively measured and self-reported weight, self-reported BMI, or the percentage value of the inaccurate estimation of BMI. 

We performed univariate and multivariate logistic regression analyses to predict the accuracy of self-reported BMI based on self-reported height and weight. The accuracy of the self-reported BMI was defined as the difference in objectively measured BMI equal to or smaller than ±0.25 kg/m^2^. We did not impute any missing data. All predictors were included in the multivariate logistic regression model and all effect estimates were adjusted for the other variable included in the model. Statistical significance was set at *P* values < 0.05. All analyses were performed using SPSS version 21.0 (IBM Inc., Chicago, IL, USA).

## 3. Results

### 3.1. Sociodemographic Characteristics of the Sample

Table 1 summarizes the sociodemographic characteristics of the participants. Among 531 participants, 318 were enrolled in a physical activity program and 213 participated in weight loss programs. The mean age (standard deviation (SD)) was 50.0 (SD = 12.0; range: 24–78) years. In total, 455 (85.7%) participants were women, and 388 (73.1%) participants were in paid employment. The study population was diverse: 218 (41.1%), 146 (27.5%), 98 (18.5%), 33 (6.2%), and 36 (6.8%) participants were Caucasian, Asian, Hispanic, African American, and mixed-race/other, respectively. Notably, 198 (37.3%) participants had a gym membership but were currently inactive based on the Stanford Brief Activity Survey [16]. Regarding cardiovascular risk factors, 153 (28.8%), 170 (32.0%), and 75 (14.7%) had high blood pressure, high cholesterol levels, and a family history of heart attack, respectively.

### 3.2. Self-Weighing and Knowledge of BMI

In total, 409 (77.0%) participants reported having a weight scale at home (Table 1), but only 222 (41.8%) participants weighed themselves at least once a week. Moreover, 172 (32.4%) participants did not regularly self-weigh, and 128 (24.1%) reported self-weighing at least once a month. Only 123 (23.2%) participants reported that they knew their BMI, 407 (76.6%) did not know, and 1 (0.2%) response was missing. A notable finding was that 101 (25.3%) participants with obesity who did not know their BMI guessed that their BMI would be categorized in the overweight group (data not shown in Table 1).

Table 2 presents differences between objectively measured and self-reported weight and BMI by weight group quartiles. Overall, differences were observed among the four groups (*F*(3, 527) = 7.37, *p* < 0.001). Participants in the fourth quartile group had the largest underreporting of their weight (mean = 1.2 kg, SD = 3.0), whereas those in the first quartile group overreported their weight (mean = –0.6 kg, SD = 1.5). Similarly, discrepancies between the two measures increased from the first to the fourth quartile groups (*F*(3, 527) = 15.30, *p* < 0.001). Participants in the fourth quartile group underestimated their BMI (mean = 1.1 kg/m^2^, SD = 0.4) compared to participants in the first quartile group (mean = 0.2 kg/m^2^, SD = 0.8). The inaccuracy of self-reported BMI differed across the quartiles of weight groups (*F*(3, 527) = 5.308, *p* = 0.001). Moreover, 72.7%, 86.4%, 88.8%, and 86.5% of participants in the first, second, third, and fourth quartile groups, respectively, inaccurately estimated their BMI (Table 2). In summary, the results showed that, as the participants’ weight increased, they were more likely to underestimate their weight and BMI.

### 3.3. Predicting the Accuracy of Self-Reported BMI

Table 3 presents the results of the univariate logistic regression and multiple logistic regression analyses predicting the accuracy of BMI based on self-reported weight and height. When the effects of each variable were adjusted for the other variables included in the multiple logistic regression analysis, three significant predictors were identified: employment (adjusted odds ratio (AOR) = 2.138; 95% confidence interval (CI), 1.036–4.413; *p* = 0.040), high cholesterol levels (AOR = 2.192; 95% CI, 1.223–3.930; *p* = 0.008), and objectively measured weight (AOR = 0.971; 95% CI, 0.953–0.991; *p* = 0.004). For individuals with paid employment, the likelihood of an accurate prediction of BMI increased by 113.8% compared with those who were unemployed. Individuals with high cholesterol levels showed a 119.2% increased likelihood of accurately estimating their BMI. Finally, for an increase in weight by 1 kg, the likelihood of an accurate prediction of BMI decreased by 2.9%. Having a scale at home and the frequency of self-weighing behaviors were not significantly associated with the accuracy of self-reported BMI (*p* > 0.05).

## 4. Discussion

The present study aimed to describe self-weighing behaviors and examine the role of sociodemographic, medical history, self-weighing behaviors, and objectively measured weight on individuals’ BMI accuracy. The results showed that 77% of participants had a weight scale at home but only 41.8% reported self-weighing at least weekly. Since the participants in the present study were highly motivated to change their lifestyle, the prevalence of weight scale ownership and regular self-weighing could be significantly higher than that in the general population. Bramante et al. reported that, although 56% of primary care patients selected from mixed- to low-income urban areas had a home scale, only 28% reported regular daily or weekly self-weighing [19]. Thus, individuals with low-income were less likely to own a scale at home and engage in self-weighing than their counterparts. In contrast, in another online anonymous survey study, 560 (out of 33,839) primary care patients responded to the survey, and 35% and 24% of the patients reported self-weighing weekly and daily, respectively [19]. The primary sample of this online survey study was Caucasian, with at least a college degree. These findings highlight the disparities in self-weighing behaviors and the ownership of home scales.

Consistent with previous systematic reviews [3,4,5,6], the overestimation of height and the underestimation of weight, resulting in BMI underestimation, were observed in the present study. An important finding is that these discrepancies became significantly more prominent as participants’ objectively measured weight increased. In the multivariate logistic regression analysis, this finding was confirmed even after controlling for all the other potential confounding factors of sociodemographics, medical history, and self-weighing behavior that were included in the model. In several previous studies, an increase in reporting errors of weight and BMI among individuals who were overweight and obese was also observed compared to those with normal weight [3,5,6].

Unlike most previous studies, we further identified factors associated with the accuracy of BMI based on self-reported height and weight. After controlling for other factors included in the regression model, we observed that employment status, high cholesterol levels, and low objectively measured weight were significant. A previous study also found that unemployed women were more likely to underreport their BMI than employed women [20]. In the 2015 National Health Interview Survey, approximately half of the employers were offered workplace health promotions [21]. Thus, employees might have more opportunities to assess and evaluate their height, weight, and BMI at the workplace. Similar reasons can also explain the finding of high cholesterol levels. The 2021 ACC Expert Consensus Decision Pathway on the Management of Atherosclerotic Cardiovascular Disease Risk Reduction in Patients with Persistent Hypertriglyceridemia continued to emphasize lifestyle interventions such as regular physical activity and weight loss as the first line of treatment for the management of individuals with high dyslipidemia [22]. Thus, primary healthcare providers might frequently inform participants with high cholesterol levels of their BMI and lifestyle change status.

An unexpected finding was that owning a weight scale at home and frequently self-weighing were not significantly associated with the accuracy of self-reported BMI. These findings might be related to psychological (e.g., anxiety, stress regarding one’s weight and BMI, eating concerns) [23,24], social (e.g., weight stigma, the social pressure of body image) [24], and health-related factors (e.g., physical activity) [25], leading to an inaccurate estimation of weight and height. For example, women with significant eating and weight concerns [23] or with little physical activity [3] were more likely to report inaccurate weight than their counterparts. These unexpected findings may have important clinical and research implications. Previous research suggests that providing a welcoming healthcare environment to patients with obesity could alleviate negative perceptions associated with one’s weight (e.g., weight stigma) [26]. Thus, adopting a patient-centered approach that is less threatening for patients with obesity could help alleviate patients’ stress regarding their weight, BMI, and social pressure on body image, and potentially increase the accuracy of self-reported BMI. 

## 5. Strengths and Limitations

The strengths of this study were the inclusion of adults with racial and ethnic diversity, detailed information on self-weighing behaviors, and the examination of factors significantly associated with the accuracy of BMI based on self-reported height and weight. Despite these strengths, this study had some limitations. First, the present study used a cross-sectional analysis; thus, causal relationships could not be determined. Second, the study participants were motivated to participate in a lifestyle modification program. Therefore, self-weighing behaviors and self-reported weight and height accuracy could be greater than those of the general public. Moreover, due to the unequal gender group representation, the results may be more reflective of women than men. Lastly, this study did not examine negative weight-related attitudes and beliefs such as weight stigma and negative body image that may be significant predictors of internalized weight bias [27].

## 6. Conclusions

This study aimed to describe self-weighing behaviors and identify significant predictors of BMI accuracy in a diverse sample of community-dwelling adults. A number of influencing factors leading to BMI accuracy were identified. It was revealed that the accuracy of BMI was significantly associated with employment status, high cholesterol, and low objectively measured weight after controlling for potential confounding factors. In addition, discrepancies in BMI accuracy became significantly more prominent as participants’ objectively measured weight increased. With globally rising obesity rates, the accurate estimation of BMI is essential for effective obesity prevention. This study suggests that healthcare providers and public health efforts to prevent obesity prevalence would benefit from acknowledging the multi-factors (sociodemographic, medical, lifestyle status) that influence the accuracy of individuals’ BMI estimations.

## Figures and Tables

**Table 1:** Sociodemographics, cardiovascular risk factors, self-monitoring behaviors, weight, height, and body mass index (BMI) of the sample (*n* = 531).

Sociodemographics		Mean (SD) [Range] or % (*n*)
Age (years)	-	50.0 (12.0) [24–78]
Sex	Men	14.3 (76)
	Women	85.7 (455)
Education	Completed high school and some college education	24.7 (131)
	Completed college or graduate school	75.3 (400)
Marital status	Married/cohabitating	50.1 (266)
	Never married/divorced/widowed	49.9 (265)
Employment	Employed for paid	73.1 (388)
	Unemployed/home maker/retired/disabled/other	26.9 (143)
Ethnicity	White	41.1 (218)
	Black or African American	6.2 (33)
	Hispanic or Latino	18.5 (98)
	Asian	27.5 (146)
	Mixed/Other	6.8 (36)
Gym membership	Yes	37.3 (198)
How would you rate your general health status?		4.84 (1.1) [2–7]
Have you used a step counter/pedometer?	Yes	48.8 (259)
Physical activity study participation	Yes	59.9 (318)
Weight-loss study participation	Yes	40.1 (213)
**Cardiovascular risk factors**		
Told I have high blood pressure	Yes	28.8 (153)
	No	70.8 (376)
	Don’t know	0.4 (2)
Told I have high total cholesterol	Yes	32.0 (170)
	No	54.8 (291)
	Don’t know	13.2 (70)
Has your mother, sister, father, or brother had a heart attack?	Yes	14.7 (75)
**Self-weighting behaviors**		
Have a weight scale at home ^a^	Yes	77.0 (409)
How often do you weigh yourself? ^b^	Never or not regularly	32.4 (172)
	At least once a month	24.1 (128)
	At least once a week	41.8 (222)
Do you know your BMI?	Yes	23.2 (123)
IF YES, what is your BMI		30.0 (5.3) [18–50]
IF NO, do you think your BMI would be? ^c^	Underweight (<18.5 kg/m^2^)	0.2 (1)
	Normal weight (≥18.5 to <25 kg/m^2^)	12.6 (67)
	Overweight (≥25 to <30 kg/m^2^)	43.7 (232)
	Obese (≥30 kg/m^2^)	19.2 (102)
**Self-report**		
	Weight (kg)	81.6 kg (17.6) [44.5–133.8]
	Height (cm)	164.8 cm (7.7) [142.2–188.0]
	BMI (kg/m^2^)	30.0 (5.6) [18.2–50.7]
**Objective measure**		
	Weight (kg)	82.2 kg (18.1) [45.7–143.3]
	Height (cm)	163.6 cm (7.5) [144.0–188.2]
	BMI (kg/m^2^)	30.6 (5.9) [18.5–54.1]
**Difference (objective measure: self-report)**		
	Weight (kg)	0.5 kg (2.4) [−8.6–17.0]
	Height (cm)	−1.2 cm (1.9) [−7.2–8.3]
	BMI (kg/m^2^)	0.6 (1.2) [−6.8–7.0]

^a^ 9 missing cases, ^b^ 1 missing case, ^c^ 7 missing cases.

**Table 2:** Mean, standard deviation, and range of differences in objectively measured and self-reported weight and body mass index (BMI) by objectively measured weight groups (*n* = 531).

	Quartile of Objectively Measured Weight Groups				
	1st Quartile (45.7–67.8 kg)	2nd Quartile (67.9–79.9 kg)	3rd Quartile (80.0–94.1 kg)	4th Quartile (94.2–143.3 kg)	*p*-Value
Differences between objectively measured and self-reported weight (kg)	−0.6 (1.5) ^a,b^ [−5.7–4.5]	0.2 (2.1) ^c^ [−8.6–5.2]	0.7 (2.6) ^a^ [−7.7–9.7]	1.2 (3.0) ^b,c^ [−6.7–17.0]	<0.001
Differences between objectively measured and self-reported BMI (kg/m^2^)	0.2 (0.8) ^d,e^ [−1.8–2.4]	0.4 (1.1) ^f,g^ [−6.8–2.5]	0.8 (1.2) ^d,f^ [−3.6–4.3]	1.1 (1.4) ^e,g^ [−2.6–7.0]	<0.001
Inaccuracy of self-reported BMI (% accurate within group)	72.7% ^h,i,j^	86.4% ^h^	88.8% ^i^	86.5% ^j^	0.001

Note. Self-reported BMI was calculated based on individuals’ self-reported height and weight. ^a^ *p* = 0.039; ^b^ *p* < 0.001; ^c^ *p* = 0.004; ^d^ *p* < 0.001; ^e^ *p* < 0.001; ^f^ *p* = 0.030; ^g^ *p* < 0.001; ^h^ *p* = 0.016; ^i^ *p* = 0.002; ^j^ *p* = 0.014.

**Table 3:** Predicting the accuracy of body mass index (BMI) (kg/m^2^) based on self-reported weight and height.

		Unadjusted			Adjusted ^a^		
		OR	95% CI	*p* Value	OR	95% CI	*p* Value
Age		0.999	0.980–1.019	0.943	0.995	0.967–1.022	0.696
Female Sex		0.846	0.450–1.592	0.605	0.774	0.310–1.930	0.582
Completed college or graduate school		1.698	0.936–3.078	0.081	1.558	0.748–3.244	0.237
Married/cohabitating		1.509	0.947–2.403	0.083	1.233	0.711–2.139	0.456
Employed		1.284	0.747–2.205	0.366	2.138	1.036–4.413	0.040
Ethnicity	White (ref)	1		0.657	1		0.951
	Black or African American	0.653	0.217–1.967	0.449	1.049	0.264–4.171	0.946
	Hispanic or Latino	0.924	0.487–1.753	0.809	0.939	0.383–2.304	0.891
	Asian	1.026	0.592–1.778	0.926	0.754	0.359–1.585	0.456
	Other ^b^	0.431	0.126–1.477	0.180	0.821	0.220–3.058	0.768
Gym membership		1.160	0.725–1.856	0.535	1.002	0.569–1.764	0.993
General health status		0.999	0.810–1.233	0.994	0.857	0.654–1.122	0.262
Pedometer use		1.090	0.688–1.726	0.714	0.919	0.530–1.595	0.765
High blood pressure		1.228	0.748–2.017	0.417	1.187	0.632–2.227	0.594
High cholesterol		1.971	1.190–3.263	0.008	2.192	1.223–3.930	0.008
Family history of heart attack		1.160	0.617–2.180	0.645	1.548	0.751–3.191	0.237
Weight loss program ^c^		1.006	0.629–1.607	0.981	1.010	0.480–2.127	0.979
Has a scale at home		1.379	0.768–2.474	0.282	1.601	0.745–3.443	0.228
Self-monitoring of weight	Never or don’t regularly weigh (ref)	1		0.639	1		0.838
	Weigh once a month	1.187	0.651–2.164	0.576	1.028	0.495–2.134	0.941
	Weigh once a week	0.898	0.519–1.554	0.700	0.851	0.428–1.694	0.646
Objectively measured weight (kg)		0.981	0.967–0.994	0.006	0.971	0.953–0.991	0.004

^a^ All effect estimates were adjusted for the other variable included in the regression model. ^b^ The ‘Other’ category includes Native Hawaiian/Pacific Islander and multiracial individuals. ^c^ The reference group was in a physical activity intervention program.

## Data Availability

Not applicable.
